# Dehydroepiandrosterone Cocrystals with Improved Solubility and Bioavailability

**DOI:** 10.3390/pharmaceutics14112478

**Published:** 2022-11-16

**Authors:** Yihua Jiang, Yinxiang Cheng, Mengyuan Xia, Bingrui Zhang, Qiaoce Ding, Liye Lu, Jian-Rong Wang, Xuefeng Mei

**Affiliations:** 1 Pharmaceutical Analytical & Solid-State Chemistry Research Center, Shanghai Institute of Materia Medica, Chinese Academy of Sciences, Shanghai 201203, China; 2 University of Chinese Academy of Sciences, No. 19A Yuquan Road, Beijing 100049, China

**Keywords:** DHEA, cocrystallization, crystal structure, solubility, bioavailability

## Abstract

Dehydroepiandrosterone (DHEA) is an FDA-approved food supplement used as an assisted reproductive sex hormone. The bioavailability is severely limited by its poor solubility (23 µg/mL). Herein, we aimed to modulate its solubility through cocrystallization. Eight cocrystals of DHEA with pyrocatechol (CAT), hydroquinone (HQ), resorcinol (RES), phloroglucinol (PG), 1,5-dihydroxy naphthalene (DHN), p-hydroxybenzoic acid (PHBA), gallic acid (GA), and 5-hydroxyisophthalic acid (5HIPA) were designed and synthesized. Some basic characterization tools, including powder X-ray diffraction, thermogravimetric analysis, differential scanning calorimetry, and Fourier transform infrared spectroscopy, were also applied in our work for basic analyses of cocrystals. It is indicated that DHEA-GA exhibits its superiority in dissolution and pharmacokinetic behaviors. While the area under the curve values of DHEA-GA is improved at the ratio of 2.2, the corresponding bioavailability of DHEA is expected to be accordingly increased.

## 1. Introduction

**Scheme 1:**
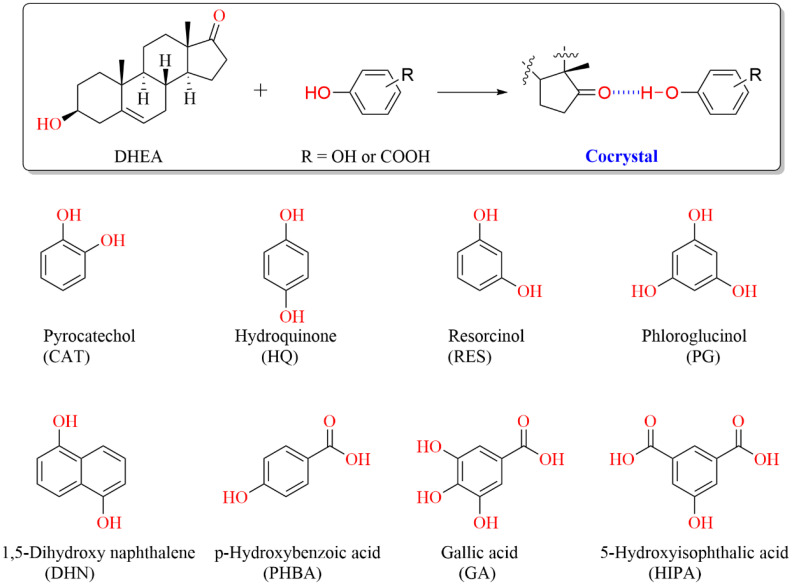
The chemical structures of DHEA and coformers.

Diminished ovarian reserve (DOR) is the third leading cause of infertility after multiple factors and endometriosis at a ratio of 13.0% [1]. In a great deal of clinical in-vitro fertilization (IVF) tests, the overall clinical pregnancy rate of women with DOR increased significantly as well as miscarriage rate decreased to some extent after treatment with dehydroepiandrosterone (DHEA, Scheme 1) [2,3]. Though the accurate mechanism of this phenomenon is not clear, it’s generally recognized that the downstream androgen of DHEA, like testosterone, acts as an important factor in this process. In detail, as the plasma concentration of testosterone and dehydroepiandrosterone sulfate (DHEA-S) rises, so does that of the anti-Müllerian hormone (AMH) [1,4]. Moreover, it seems that the higher the plasma concentration of AMH, the higher the clinical pregnancy rate of IVF cycles [5,6]. Besides, there is another consensus that the secretion of insulin-like growth factors-1 (IGF-1) can be further promoted after taking DHEA, improving the quality of follicles and oocytes [7,8]. DHEA has been utilized at nearly one-third of IVF centers worldwide before the superovulation process [9]. In this way, the requirement for further improved plasma concentration of DHEA is extremely urgent for women who undergo the IVF process. 

DHEA is a functional food supplement in the USA and has been applied as an assisted reproductive sex hormone in recent decades. It’s an endogenous precursor for androgen and estrogen to assist in regulating the endocrine system and improving the physiological state of gonadal tissue [10]. However, like other steroid hormones, DHEA is poor-aqueous soluble (23 µg/mL) and exhibits the first-pass effect through the oral-administration route, which leads to its poor absolute bioavailability (only 3.1% in the rhesus monkey) [11]. Currently, some feasible methods, including nanotechnology [12], cyclodextrin embedding method [13], cocrystallization [14], amorphous solid dispersion technology [15], and other novel techniques, have been proposed to improve the solubility of poor-solubility drugs. A cocrystal is generally defined as a single crystalline phase including two or more different molecules at a stoichiometric ratio in a certain lattice [16]. Due to the various physicochemical and biological properties of coformers, cocrystallization is often regarded as a flexible and effective solution to modulate the properties of active pharmaceutical ingredients (API) [17]. Additionally, cocrystals can often exhibit better dissolution performance in-vitro and in-vivo compared to its counterpart owing to the non-covalent interactions between the API and aqueous-soluble coformers [18,19]. 

Since 1987, when Donald J. Cram, Jean-Marie Lehn, and Charles J. Pedersen were awarded the Nobel Prize in Chemistry for their development and use of molecules with structure-specific interactions of high selectivity, the concept of supramolecular chemistry was developed and widely applied as a cross-discipline in many fields like organic chemistry, physical chemistry, coordination chemistry, material sciences, biological science, and pharmaceutical chemistry [20,21]. Supramolecular chemistry, which is different from traditional chemistry, mainly focuses on the noncovalent interactions between two or more chemical species during the construction of complex but organized entities, including hydrogen bonds, dipole–dipole interactions, Van der Waals, π···π interactions, dispersion interactions, and so on [20,21]. As Jean-Marie Lehn described, supramolecular chemistry is an “information science” which contains the instruction set to create the large and complex assembly in its constituent components [22]. Furthermore, crystal engineering is regarded as the major endeavor in solid-state supramolecular chemistry, mainly applied to design molecular crystals with various physical and chemical properties [23]. Supramolecular synthon is the basic and spatial arrangement of intermolecular interactions in crystal engineering [24]. Supramolecular synthon was exactly defined by Desiraju in 1995 as “the structural units within supermolecules which can be formed and/or assembled by known or conceivable synthetic operations involving intermolecular interactions” [25]. According to the structure of two (or more) function groups, supramolecular synthon is classified as homosynthon between similar functional groups and heterosynthon between different ones [26]. As aforementioned, noncovalent interactions play important roles in supramolecular chemistry. Among them, the hydrogen bond stands out due to its high directionality and strength, which makes the prediction and control of molecular orientation more reliable [26]. According to Cambridge Structure Database (CSD), many structures are constructed based on hydrogen bonds like N···H-O, O···H-O, and N···H-N hydrogen bonds [26]. In addition, the prediction and design of hydrogen bonds have also been widely used in crystal structure prediction (CSP) [27]. According to the theory of crystal engineering, the design of a supramolecular synthon of cocrystals has been regarded as an effective way to tune the specific physicochemical properties of API based on hydrogen bonds. 

According to its steroid-based four-linked cyclopentanophenanthrene ring structure with a hydroxyl group at ring A and a ketone carbonyl group at ring D, we can see that the C=O···HO-Ar heterosynthon can be the most competitive synthon in cocrystallizing steroids. Therefore, a series of phenolic compounds as coformers were selected for cocrystal construction. Ultimately, crystallization attempts resulted in eight novel DHEA cocrystals: DHEA-CAT, DHEA-HQ, DHEA-RES, DHEA-PG-DH, DHEA-DHN, DHEA-PHBA, DHEA-GA, and DHEA-5HIPA (Scheme 1). Comprehensive analyses of their structures and physicochemical properties, with special emphasis on improving their solubility, are presented. Notably, powder dissolution profiles show that DHEA-GA exhibits a higher solubility than other cocrystals and crystalline DHEA, which leads further to greatly improved bioavailability.

## 2. Materials and Methods

### 2.1. Materials

DHEA (Form I) [28] (purity > 99%) was purchased from Shanghai Macklin Biochemical Co., Ltd., Shanghai, China. while coformers (purity > 98%) and polymers (purity > 98%) were both obtained from Shanghai Aladdin Biochemical Technology Co., Ltd., Shanghai, China. All solvents at analytical grade and bovine serum albumin (BSA) (purity > 98%) were bought commercially from Sinopharm Chemical Reagent Co., Ltd., Shanghai, China. All chemicals were used without further purification in this study. Besides, the pH 2.0 buffer in this study consisted of 0.2 mol/L hydrochloric acid and 0.2 mol/L phosphate. In contrast, in the pH 4.5 solution, hydrochloric acid was replaced by citric acid monohydrate to adjust the pH value of this system. As for the pH 6.8 buffer, it was made up of 0.2 mol/L phosphate solution.

### 2.2. Preparation of DHEA Cocrystals

Liquid-assisted grinding, slurry, and slow cooling are common methods to prepare multi-component crystals [29,30]. Most cocrystals of DHEA were obtained through the solid-state method. About thirty coformers were selected and screened, as shown in Table S1. In detail, a certain stoichiometric ratio of DHEA (0.1 mmol) and coformer (0.1 mmol) were added into a grinding tube with 10 µL methanol in it. Then three or five milling balls at 1 mm were placed into the grinding tube before the grinding procedure started. After thirty minutes of milling at the frequency of 40 Hz with a Retsch MM200 apparatus, the desirable cocrystal was obtained. Moreover, some cocrystals were obtained via a slurry method, such as DHEA-RES and DHEA-DHN. About 50 mg DHEA and 50 mg coformer were added into 1 mL mixture solvent (consisting of 20% ethyl acetate and 80% hexane) and stirred overnight at 500 rpm under room temperature. Finally, the cocrystal sample was obtained after further filtration and vacuum drying. A slow cooling method was also applied to prepare the cocrystal of DHEA and phloroglucinol (PG). About 144 mg DHEA and 63.1 mg PG at the stoichiometric ratio of 1:1 was added into 4 mL acetonitrile at first, and the resulting clear solution was cooled at −20 °C for two weeks. Consequently, the bulk transparent cocrystal of DHEA-PG-DH was collected. All powder samples of obtained cocrystals after vacuum drying were confirmed by PXRD.

### 2.3. Preparation of Single Crystals

Most single crystals of DHEA cocrystals were obtained through slow evaporation at ambient temperature. About 20 mg of pure cocrystal sample was dissolved into the mixed solution consisting of 60% acetone and 40% n-hexane. After slow evaporation for 3–4 days at room temperature, the transparent single crystals were obtained and further analyzed using a Bruker D8 Venture diffractometer. This method was feasible to gain the single crystal of DHEA-RES, DHEA-CAT, DHEA-HQ, DHEA-GA, DHEA-PHBA, and DHEA-5HIPA cocrystals. A cooling method was applied for the other two cocrystals to gain their single crystals. As for DHEA-DHN, about 20 mg sample was added into 1 mL of ethyl acetate (70%) and n-hexane (30%), and the generated clear solution was kept at −20 °C for a few days. The single crystal was finally available under this condition. As regards the DHEA-PG-DH cocrystal, about 200 mg sample of DHEA and PG at a stoichiometric ratio of 1:1 was dissolved in 4 mL acetonitrile. Then the single crystal of DHEA-PG-DH was available under a slow crystallization process for half a month.

### 2.4. Powder X-ray Diffraction (PXRD)

PXRD patterns were collected on a Bruker D8 Advance X-ray diffractometer with Cu Kα radiation. The voltage and current of the generator were set to 40 kV and 40 mA, respectively. The scanning range was 3–40° 2θ with the scanning speed at 0.1 s/step under the ambient temperature. Then the data was imaged and integrated with the help of RINT Rapid, while the final peak analysis was conducted using Jade 6.0 from Rigaku.

### 2.5. Single Crystal X-ray Diffraction (SCXRD)

All X-ray diffraction collections of the single crystal sample were performed on a Bruker D8 Venture diffractometer with Mo Kα radiation (λ = 0.71073 Å) under 150 K, or 170 K. Intensity data were integrated and scaled on Program SAINT. At the same time, this was influenced by the adsorption effect and was further refined and adjusted using SADABS. The Olex 2 software was utilized to solve and refine the crystal structures of all multi-component forms. The crystal structures were solved directly on SHELXTL and further refined with the full-matrix least-squares technique on SHELX-2017 software. All non-hydrogen atoms were refined with isotopic atomic displacement parameters, while all hydrogen atoms were located in calculated positions and subsequently refined with the riding model. Crystallographic data in cif format has been deposited in the Cambridge Crystallographic Data Center, CCDC N0 2212357-2212364. 

### 2.6. Differential Scanning Calorimetry (DSC)

All the experiments were carried out on a DSC Q2000 instrument (TA Instruments, New Castle, DE, USA) with the nitrogen gas flow rate at 50 mL·min^−1^. About 5 mg sample in a sealed non-hermetic aluminum pan was heated at the heating rate of 10 °C·min^−1^. The instrument was calibrated by the two-point calibration using indium and tin.

### 2.7. Thermogravimetric Analysis (TGA)

Thermogravimetric analysis was performed on a TGA 55 equipment (TA Instruments, New Castle, DE, USA). The nitrogen was applied as purge gas at 20 mL·min^−1^. The sample was placed in an open aluminum oxide pan and heated at the rate of 10 °C·min^−1^ from 25 °C to 410 °C.

### 2.8. Fourier Transformation Infrared (FT-IR) Spectroscopy

The cocrystal sample was dispersed in KBr and scanned at a resolution of 4 cm^−1^ in the range from 4000 to 400 cm^−1^ using a ThermoFisher Nicolet iS50 FT-IR spectrometer. For each spectrum, thirty-two scans were acquired.

### 2.9. Solubility and Powder Dissolution Tests

Considering the effect of particle size on the results of solubility and powder dissolution tests, all samples were sieved through 100-mesh sieves. Solubility test was carried out in pH 2.0, pH 4.5, and pH 6.8 buffer with 0.5% PVP K13-18 and water, respectively. About 20 mg sample was added into 1 mL different buffer solution and then stirred at the rate of 250 rpm for 12 h under the ambient temperature. Then the suspension was centrifuged at 12,000 rpm for 5 min. The supernatant was filtered through the 0.45 µm nylon filter and finally submitted to HPLC analysis, while the PXRD pattern of residue powder after vacuum drying was further collected.

A mini-Bath dissolution device equipped with a Julabo-5 heater/circulator was applied for powder dissolution experiments. The experiment temperature was set at 37 °C, and the rotation speed was 25 rpm. About 50 mg sample was placed in a container with 15 mL buffer. Subsequently, about 0.5 mL sample was collected and filtered at different time points through a 0.45 µm nylon filter and then analyzed by HPLC. The collection time points were set at 5, 15, 30, 60, 90, 120, and 180 min. The residue powder in the container was filtered and dried for PXRD analysis.

### 2.10. In-Vivo Pharmacokinetic Experiments in Rat

All pharmacokinetic experiments were supported by the Institutional Animal Care and Use Committee of the Shanghai Institute of Material Medica and conformed to the Guide for Care and Use of Laboratory Animals. Six male Sprague-Dawley rats (200–250 g) were set as a group and fasted overnight with only water available before drug administration. The dosage was set as 21 mg/kg in male rats (taking the content of DHEA as equivalent). The suspension samples for oral administration were prepared by uniformly dispersing the solid samples into the solution, which contained 0.5% (wt%) CMC-Na and 0.5% (wt%) PVP K13-18. Since DHEA was an endogenous steroid hormone, the blood samples before dosing were collected as initial blank serum samples to investigate the effect of exogenous DHEA on plasma concentration. After dosing, 700 µL blood samples were collected from the orbital sinus at specific time points and placed into heparinized tubes. The time points were set at 0, 5, 10, 15, 30, 60, 120, 240, and 360 min. After centrifugation at 14,000 rpm for 10 min, supernatants of collected blood samples were transferred and stored at −80 °C until HPLC-MS/MS analysis. Additionally, the fodder was available for rats again when the blood samples of 240 min were collected.

### 2.11. HPLC Analysis

The accurate concentration of DHEA was analyzed on an Agilent 1260 series HPLC (Agilent Technologies Co., Ltd., Santa Clara, CA, USA) equipped with a quaternary pump (G1311C), a diode-array detector (G1315D) and a 4.6 × 150 mm, 5 µm Agilent ZORBAX Eclipse Plus C18 column. The injected volume was set to 50 µL, while the UV-vis wavelength was set to 210 nm. The column temperature was 35 °C. An isometric elution method using methanol and deionized water at the ratio of 75:25 (*v*/*v*) as the mobile phase was performed with the flow rate at 1 mL/min. The retention time point for DHEA was 4 min.

### 2.12. HPLC-MS/MS Analysis

Acetonitrile (ACN) was used to extract DHEA from serum and denature the protein. Firstly, ACN (400 µL) and an internal standard solution of abiraterone (250 ng/mL, 50 µL) were added to a 200 µL thawing serum sample. After thoroughly blending in an ultrasonic apparatus for 15 min, the mixture was further centrifuged for 10 min at 12,000 rpm. Then 200 µL supernatant was transferred into a 96-well plate and reacted with 400 µL hydroxylamine hydrochloride solution (0.1 mol/L) for further derivatization. The reaction was conducted at 60 °C for an hour, and the resultant solution was submitted for HPLC-MS/MS analysis directly. 

The quantification of DHEA in rat plasma after oral administration was carried out by HPLC-MS/MS. A SCIEX Triple QuadTM 4500 LC-MS instrument with a Polaris 3 C18-A column (2.0 × 50 mm) was applied to detect DHEA in plasma. The column temperature was set to 40 °C. The mobile phase consisted of A: 1% acetic acid in water and B: methanol. The gradient elution method started with 55% A and linearly decreased to 20% in 2 min, then held for 1 min, and finally returned to 55% in half a minute and held for one and a half minutes. The flow rate was set to 0.6 mL/min with the injection volume at 10 µL. The detection wavelength was 210 nm. Detection and quantification were employed in multiple reaction monitoring modes (MRM), with m/z 304.4 → 253.2 for DHEA pharmacokinetic analysis. 

GraphPad Prism 8.0 was applied for the statistical analyses. The results are significant if the *p*-value < 0.05 or less. 

### 2.13. Theoretical Calculation

Crystal Explorer 21.5 software was utilized to calculate the Hirshfeld Surface of DHEA cocrystals [31]. For Molecular Electrostatic Potential surfaces (MEPs) analysis, the molecular structures of DHEA and coformers were optimized by Gaussian 16 at the B3LYP/6-31G++ (d, p) level of theory, and then the single point energy calculation was conducted at the DFT/B3LYP with 6-311G++ (d, p) basis set to obtain the wave function files. During the calculation process, the ‘empirical dispersion = gd3bj’ was added to the keywords. In the end, Multiwfn 3.8 was applied for MEPs analysis, while the visualized results were obtained by Visual Molecular Dynamics (VMD) 1.9.3 [32,33].

## 3. Results and Discussion

### 3.1. Design of DHEA Cocrystals

DHEA is a steroid hormone molecule with a hydroxyl group and a ketone carbonyl group. From the crystal structure of DHEA, it can be seen that the alcohol hydroxyl group and the ketone carbonyl group on it form a tight and regular 1D chain in a head-to-tail manner (Figure S1). To break this rigid interaction, strong H-bond donors on rigid structures were selected for cocrystal screening, like phenol hydroxyl groups, benzoic acid groups, and aniline groups (Table S1). MEPs analysis is also applied to visualize the electrostatic potential distribution of coformers and DHEA, which is regarded as a measure of the probability of forming a cocrystal between a specific coformer and DHEA.

**Figure 1:**
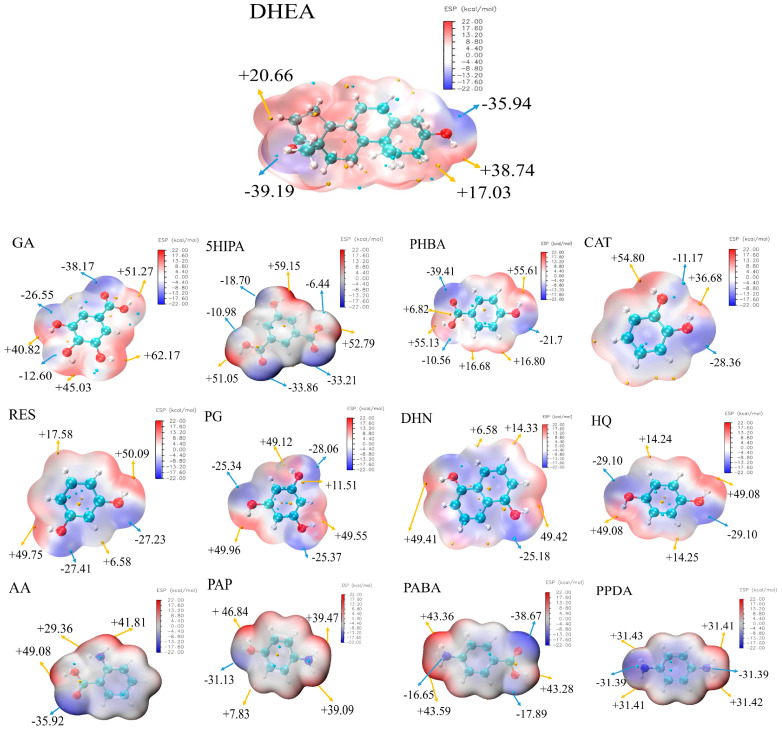
MEPs mapped onto 0.001 a.u. electron density isosurface of DHEA and twelve coformers. The electrostatic potential extreme points (kcal/mol) are represented in blue and red, and the value is marked in black.

Twelve typical coformers are chosen and calculated for MEPs analyses (Figure 1). Via visualization tools, the charge distributions on the surfaces of DHEA and coformers are displayed in Figure 1, where the positive and negative areas are shown in red and blue, respectively. The values of extreme points (marked in black in Figure 1) are also summarized in Table 1. According to Etter’s rules, the best H-bond donors are paired with the best acceptors [34]. This “rule of thumb” is further refined and applied to predict the matching site of specific H-bond donors and acceptors and rank synthons for crystal engineering [35]. Comparing MEPs values of functional groups on coformers and DHEA, it is supposed that hydroxyl groups on coformers are more electropositive and tend to act as H-bond donors during the formation of cocrystals. In contrast, the ketone carbonyl group on DHEA prefers to act as an H-bond acceptor with the most negative MEPs value at −39.19 kcal/mol. Moreover, it is supposed that the ketone carbonyl group on DHEA prefers to act with the H-bond donor that possesses a higher positive MEPs value. In addition, twelve coformers are ranked based on their extremely positive MEPs value on their surface in Table 1. Table 1 shows that the top eight coformers can form cocrystals with DHEA successfully via keto-phenol supramolecular synthon, which also fits our theory well (Scheme 1). In particular, although anthranilic acid (AA) possesses the same positive MEP values as hydroquinone (HQ), no cocrystal formation between DHEA and AA was found. It might be attributed to the through-space effect, which considers the effect of neighboring atoms in a functional group in forming H-bond interactions. When the ketone carbonyl group interacts with the phenol hydroxyl group, a long-range through-space attractive interaction is also found between the carbonyl group and the adjacent aromatic C-H groups of a phenol hydroxyl group. However, the corresponding interaction is repulsive for a carboxylic group [36].

### 3.2. PXRD Analysis

During the screening process, PXRD analysis is considered a predominant method to detect the formation of a new multi-component phase and identify the purity of the new solid substance. All experimental and simulated PXRD patterns of these eight cocrystals are exhibited in Figure S2a–h, which are different from either that of DHEA or the corresponding coformers. Taking DHEA-GA as an example, the appearance of new characteristic peaks at 2θ = 9.78°, 10.24°, 16.58°, and 17.50° suggests the generation of a new phase, while the characteristic peaks of DHEA at 2θ = 7.89°, 14.85°, 15.38° and that of gallic acid at 2θ = 8.09°, 16.18°, and 19.14° disappear at the same time. Besides, all the patterns of powder samples are closely matched with the simulated patterns obtained from the single crystal diffraction data, which also proves the formation of a new phase with high purity.

### 3.3. Single Crystal Structure

**Figure 2:**
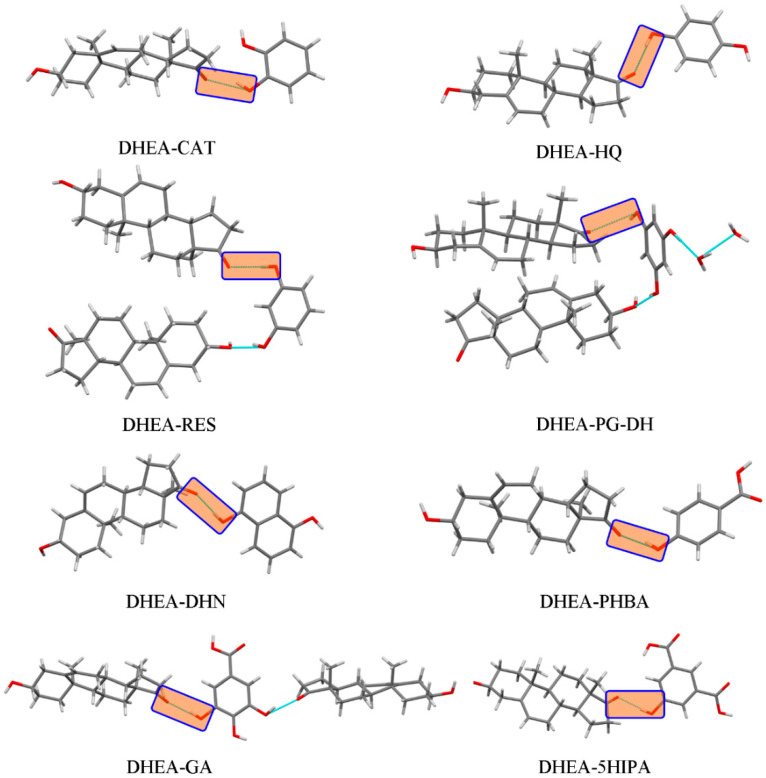
ASU of DHEA cocrystals with C=O···HO-Ar synthon highlighted.

The key crystallographic parameters of DHEA cocrystals are summarized in Table S2. In summary, as we supposed in Scheme 1, rigid phenolic compounds insert into the head-to-tail arrangement of DHEA molecules successfully in a C=O···HO-Ar mode as expected. Asymmetric units (ASU) of eight DHEA cocrystals with C=O···HO-Ar synthon are highlighted in Figure 2.

**Figure 3:**
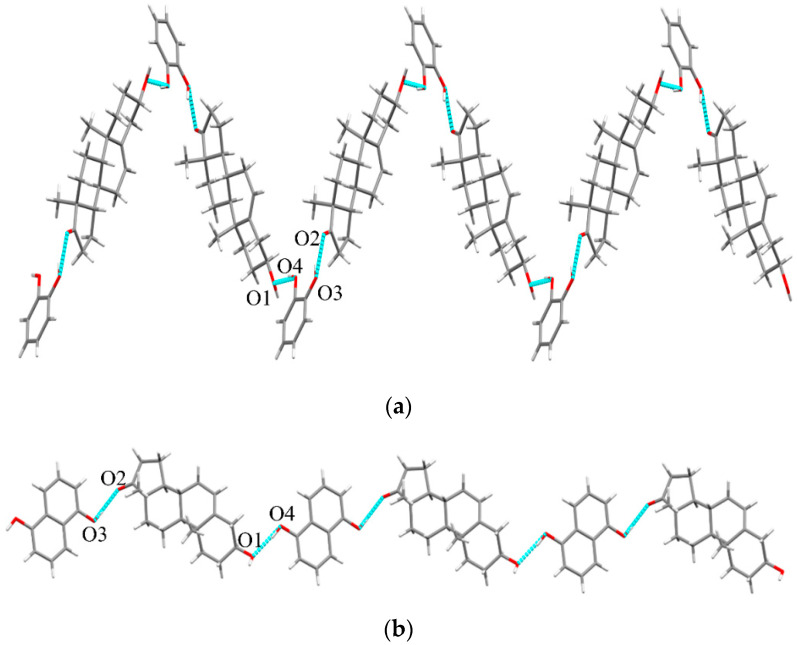
1D hydrogen-bonding interaction of (**a**) DHEA-CAT and (**b**) DHEA-DHN.

DHEA-CAT cocrystal (1:1). The DHEA-CAT crystalizes in the orthorhombic *P*2_1_2_1_2_1_ space group with Z = 4 (the Z number represents the cocrystal formula in a unit cell). The ASU of DHEA-CAT contains one DHEA molecule and one CAT molecule (Figure 2). Two molecules are connected through C=O···HO-Ar synthon O2···H3A-O3 (2.726 Å) as expected. Then 1D infinite hydrogen-bonding chain is formed via O4-H4···O1 (2.763 Å) interactions in Figure 3a.

DHEA-DHN cocrystal (1:1). The DHEA-DHN takes the orthorhombic *P*2_1_2_1_2_1_ space group with Z = 4. One DHEA molecule and one DHN molecule are presented in the ASU of DHEA-DHN. Similar to that of DHEA-CAT, DHEA and DHN molecules are connected through C=O···HO-Ar synthon O2···H3A-O3 (2.736 Å) as expected. Additionally, each DHEA-DHN unit is linked via O4-H4···O1 (2.739 Å) interaction, forming the 1D infinite hydrogen-bonding chain in Figure 3b.

**Figure 4:**
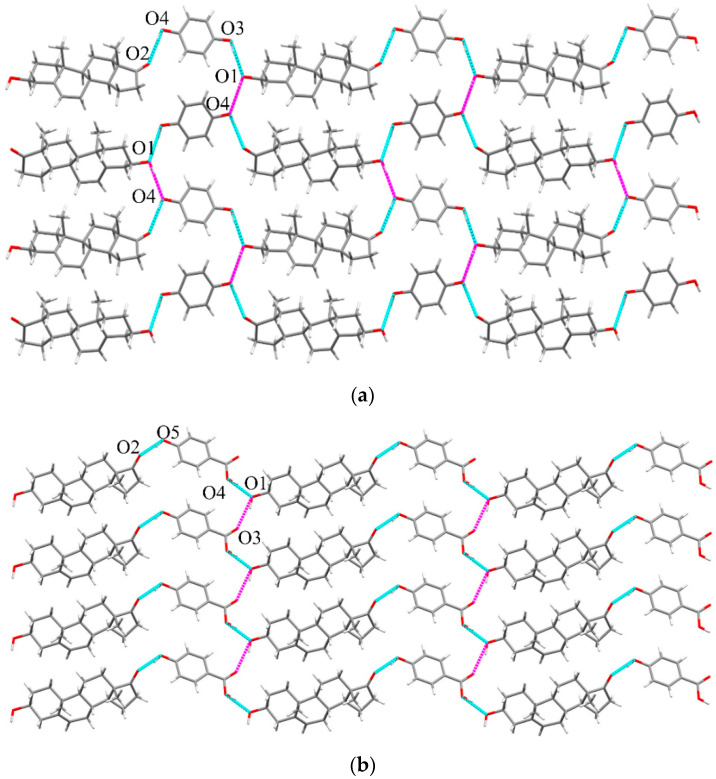
2D architecture of (**a**) DHEA-HQ and (**b**) DHEA-PHBA.

DHEA-HQ cocrystal (1:1). The ASU of DHEA-HQ contains one DHEA molecule and one HQ molecule (orthorhombic, *P*2_1_2_1_2_1_). As the way we designed (C=O···HO-Ar synthon), DHEA interacts with HQ through O2···H4-O4 (2.833 Å), forming a basic structural unit. Subsequently, DHEA-HQ unit links each other via O3-H3A···O1 (2.777 Å) interactions to form a 1D supramolecular chain, then O1-H1···O4 (2.881 Å) interaction contributes the final formation of 2D infinite chain in Figure 4a.

DHEA-PHBA cocrystal (1:1). The structure of DHEA-PHBA is very similar to that of DHEA-HQ. The space group is *P*2_1_ with Z = 2. There is one DHEA molecule and one PHBA molecule in the ASU. Analogously, C=O···HO-Ar synthon O2···H5-O5 (2.731 Å) interaction is also observed in DHEA-PHBA. To construct the 2D framework, O4-H4·· O1 (2.584 Å) interaction (blue line) and O1-H1···O3 (2.893 Å) interaction (purple line) are regarded as the bridge of infinite 1D chain and following 2D skeleton, respectively (Figure 4b).

**Figure 5:**
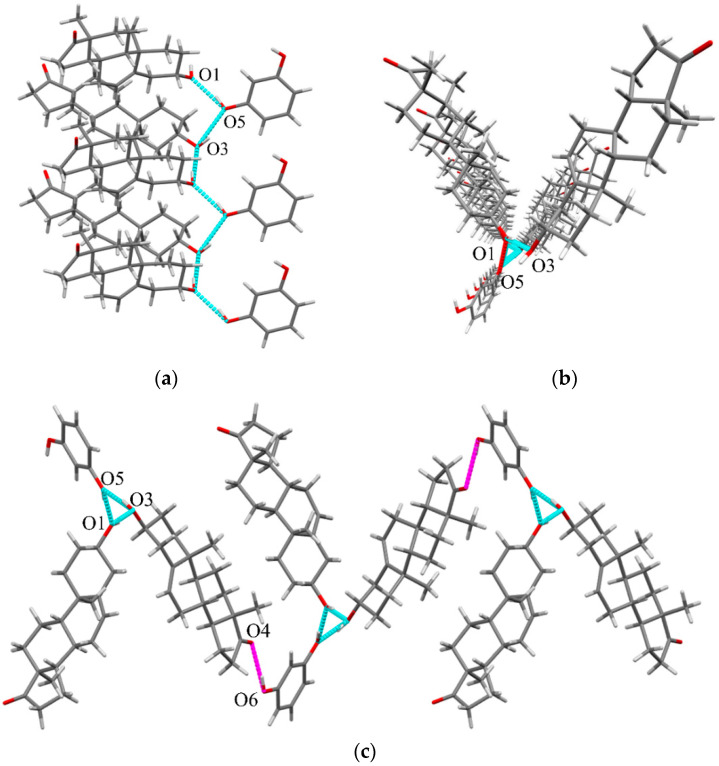
(**a**) 1D hydrogen-bonding interaction, (**b**) supramolecular three-fold helix, and (**c**) 3D architecture of DHEA-RES.

DHEA-RES cocrystal (2:1). The DHEA-RES takes the orthorhombic *P*2_1_2_1_2_1_ space group with Z = 4. The ASU of DHEA-RES contains two DHEA molecules and one RES molecule. A supramolecular three-fold helix is formed via continuous O5-H5···O1 (2.671 Å), O1-H1···O3 (2.859 Å) and O3-H3A···O5 (2.858 Å) interactions as shown in Figure 5a,b. These helixes are then connected through O4···H6A-O6 (2.798 Å) to construct the 2D hydrogen-bonding structure in Figure 5c.

**Figure 6:**
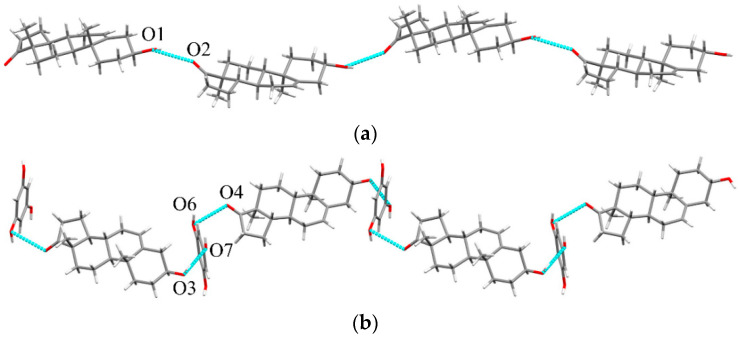

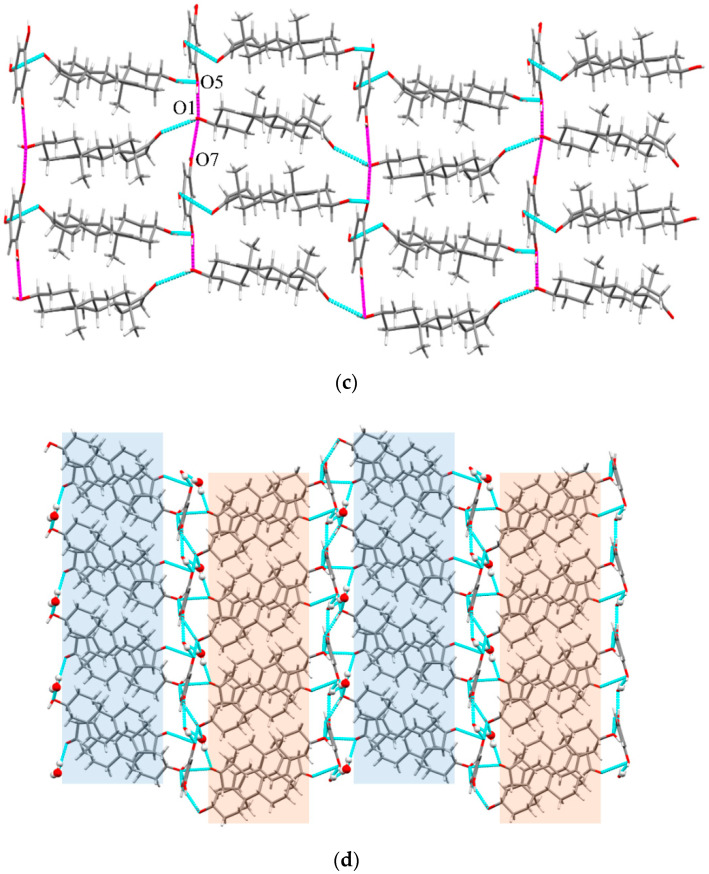
(**a**,**b**) 1D, (**c**) 2D, and (**d**) 3D supramolecular interactions of DHEA-PG-DH.

DHEA-PG cocrystal dihydrate (2:1:2). The crystal structure of DHEA-PG-DH is solved in the monoclinic *P*2_1_ space group with Z = 2. The ASU of DHEA-PG-DH contains two DHEA molecules, one PG molecule, and two water molecules. There is still a head-to-tail O1-H1···O2 (3.001 Å) interaction as that in DHEA (Figure 6a). The other 1D chain is formed by O3-H3···O7 (2.853 Å) interaction and O4···H6A-O6 (2.943 Å) interaction (Figure 6b). Two 1D chains are then connected by O7-H7···O1 (2.700 Å) interaction and O5-H5···O1 (2.817 Å) interaction to form 2D layers (Figure 6c). These 2D layers further construct the 3D supramolecular architecture by hydrogen bonds between two water molecules (Figure 6d).

**Figure 7:**
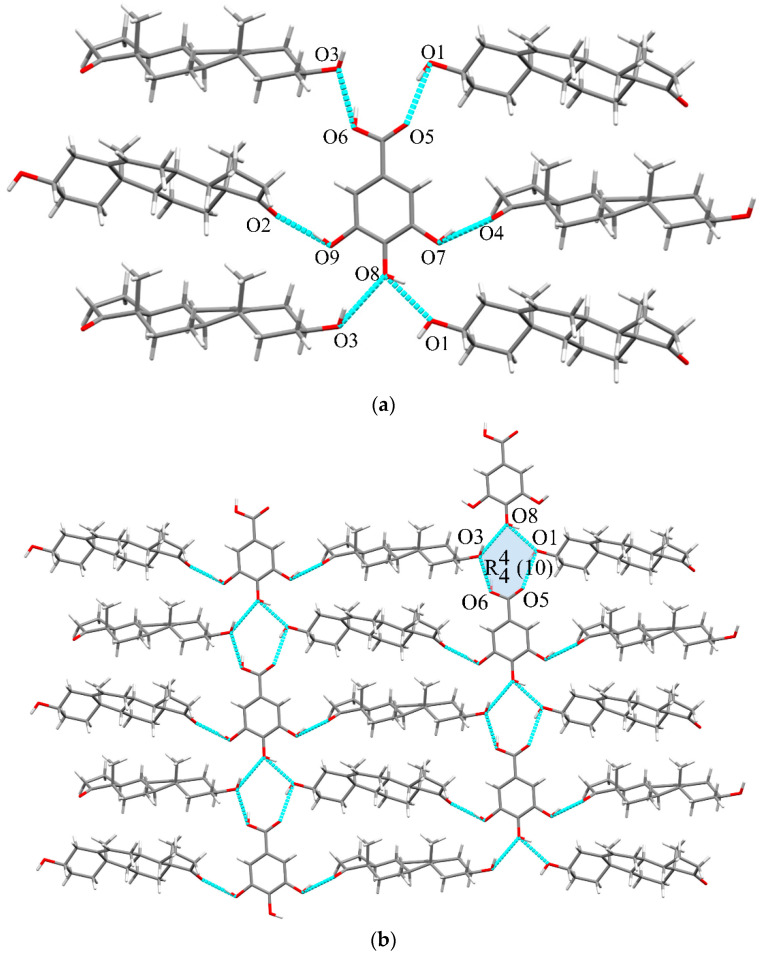
(**a**) Multi-interactions of every GA with six DHEA molecules, and (**b**) 2D supramolecular interactions of DHEA-GA along the ac plane.

DHEA-GA cocrystal (2:1). The crystal structure belongs to the monoclinic *P*2_1_ space group with DHEA and GA in 2:1 stoichiometry. Six hydrogen-bonds were formed for every GA molecule (O6-H6A···O3 (2.630 Å), O1-H1···O5 (2.763 Å), O9-H9A···O2 (2.706 Å), O7-H7···O4 (2.721 Å), O3-H3···O8 (2.984 Å) and O8-H8A···O1 (2.726 Å)) as shown in Figure 7a. No more hydrogen bonding is needed to form the final 2D supramolecular structure, where an $R_{4}^{4}$ (10) supramolecular heterosynthon was found to be an essential linker through O8-H8A···O1 (2.726 Å), O1-H1···O5 (2.763 Å), O6-H6A···O3 (2.630 Å), and O3-H3···O8 (2.984 Å) hydrogen bonding interactions (Figure 7b). Interestingly, distinct hydrophilic GA and hydrophobic DHEA areas are alternatively aligned.

**Figure 8:**
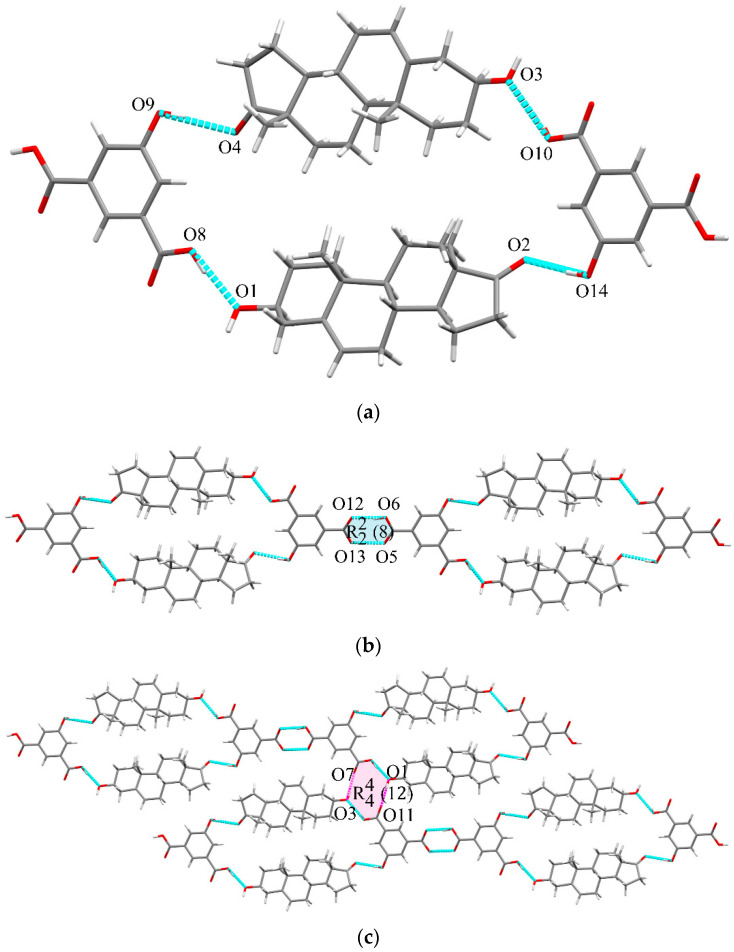
(**a**) tetramer, (**b**) 1D, and (**c**) 2D supramolecular interactions of DHEA-5HIPA.

DHEA-5HIPA cocrystal (1:1). The crystal structure belongs to the triclinic *P*1 space group with DHEA and 5HIPA in 1:1 stoichiometry. Two DHEA and two 5HIPA molecules first connected by O8−H8A···O1 (2.597 Å), O9−H9A···O4 (2.766 Å), O10−H10···O3 (2.548 Å) and O14−H14A···O2 (2.753 Å) interactions in Figure 8a. The tetramers then interact via an $R_{2}^{2}$ (8) supramolecular homosynthon through O6−H6A···O12 (2.602 Å) and O13−H13···O5 (2.586 Å) hydrogen bonds to form 1D polymeric chains (Figure 8b). Polymeric chains are further connected through an $R_{4}^{4}$ (12) supramolecular homosynthon to form 2D layers through O1−H1···O11 (2.731 Å) interaction and O3−H3A···O7 (2.701 Å) (Figure 8c).

### 3.4. Thermal Analysis

Additionally, the thermal performances of prepared samples are accessed via DSC and TGA methods to confirm the formation of new solid phases and control their quality. As for the DSC analytical result of multi-component substance, the single and sharp endothermic peak in the thermogram often indicates the formation of a single phase with high purity. Moreover, the TGA technique can also determine the content of residue solvent adsorbed to the surface or existing in the lattice. The corresponding analytical results are displayed in Figure S3a–h. Taking DHEA-PG-DH as an example, the weight loss step (4.9%) (Figure S3d) in the range of 20–120 °C indicates the loss of water accompanied by the melting process, which is consistent with its calculated content in a single lattice. 

### 3.5. FT-IR Analyses

In the FTIR spectrums of DHEA, the band at 1731 cm^−1^ is assigned to carboxyl C=O stretching vibration (Figure S5a), and the corresponding peaks of cocrystals all get red-shifted as what we summarized in Table S5. This phenomenon might be attributed to the formation of hydrogen bonds between the carboxyl group on DHEA and phenol hydrogen groups on coformers, which leads to a significant reduction in the vibration frequency of the carboxyl group. In addition, the characteristic bands of the hydrogen bond on the coformer also shift to a lower wave number direction for DHEA cocrystals. Taking GA as an example, we observed that the maximum frequency of the H-O stretch absorption shifts from 3496 cm^−1^ to 3440 cm^−1^ after acting with DHEA. These redshifts all indicated the formation of intermolecular hydrogen bonds between DHEA and coformers, which was regarded as an important indicator of the formation of a new solid form. 

### 3.6. Theoretical Calculation

**Figure 9:**
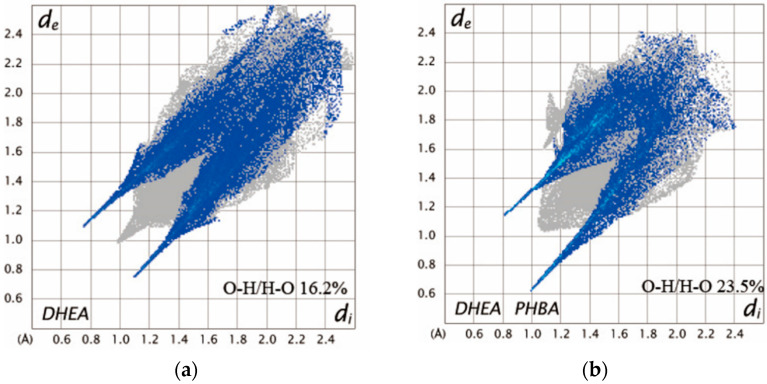

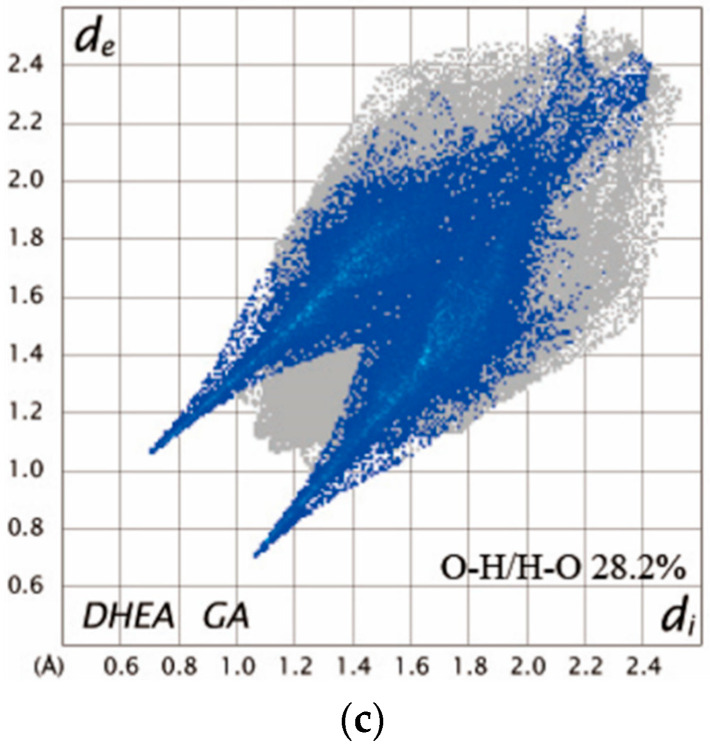
2D fingerprint plots for (**a**) DHEA, (**b**) DHEA-PHBA, and (**c**) DHEA-GA, showing the contribution of H···O/O···H interactions.

Crystal Explorer 21.5 is applied to obtain the 2D fingerprint plots of DHEA, DHEA-PHBA, and DHEA-GA as a visualization tool to encode the information qualitatively and quantitatively on intermolecular atomic close contacts. Corresponding results are presented in Figure 9. The percentage of area occupied by H-bond interactions is also marked in it. Besides, di and de are the distance from the Hirshfeld surface to the nearest nucleus inside and outside the surface, respectively. According to the literature, polar interaction (H···O) is often considered to represent hydrophilicity, while the nonpolar interactions (C···H, C···C, and C···O) to represent hydrophobicity [37]. As for DHEA-GA and DHEA-PHBA, it is obvious that the formation of an H-bond between DHEA and coformers and the construction of an H-bond net structure contributes a lot to the enhanced polar interactions between cocrystal molecules. Due to its largest proportion of H-bond interactions (28.2%) among crystalline DHEA, DHEA-PHBA, and DHEA-GA, DHEA-GA is supposed to possess the highest solubility and exhibit the best dissolution performance in-vitro. This concept is also proved by further experiments.

### 3.7. Dissolution Behavior (Solubility and Powder Dissolution)

Cocrystallization is a common method to improve the solubility and dissolution rate of poorly water-soluble drugs. During the design of cocrystals, coformers with high water solubility are often regarded as suitable components to improve the solubility of poorly soluble API molecules. It is believed that the hydrophobic API molecules can become supersaturated with the water-soluble coformer drawn out of the crystal lattice and collapse of crystal structure in the aqueous medium, which results in the enhancement of solubility [38].

**Figure 10:**
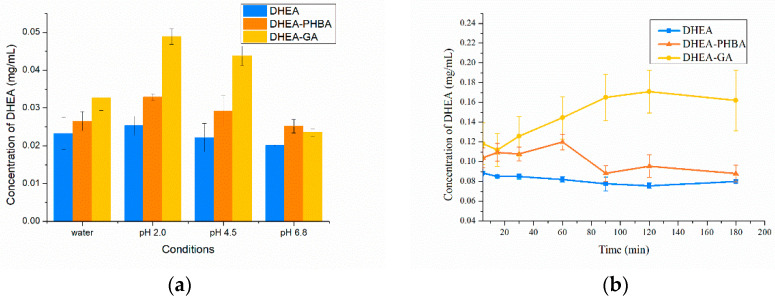
(**a**) Comparison of solubility of DHEA, DHEA-PHBA, and DHEA-GA under different conditions and (**b**) Comparison of solubility and dissolution performance of DHEA, DHEA-PHBA, and DHEA-GA under different conditions.

Based on this theory, cocrystallization is considered an effective way to enhance the solubility (23 µg/L in water) and bioavailability (3.1% in the rhesus monkey) of DHEA [11]. In view of safety, two cocrystals (DHEA-GA and DHEA-PHBA) are selected for subsequent solubility and dissolution experiments. The solubility experiments were carried out in different buffers (pH 2.0, pH 4.5, pH 6.8 buffer with 0.5% PVP K13-18, and water). Powder dissolution studies were carried out under non-sink conditions to evaluate the dissolution performances of DHEA cocrystals for further instruction about the in-vivo pharmacokinetic study. The powder dissolution experiments were performed under a pH 2.0 buffer which contained 0.5% PVP K13-18. The detailed results are summarized in Figure 10.

With the collapse of the crystal structure, the solubility of DHEA-GA and DHEA-PHBA are both improved. Compared with DHEA-PHBA, DHEA-GA exhibits greater enhancement in solubility under pH 2.0 conditions at 2-fold. As well as the results of the solubility test, DHEA-GA also exhibits its excellent advantage in enhancing the saturated solubility and maintaining the high supersaturated state of DHEA by 2-fold for three hours under powder dissolution experiments. These results are also in accord with that of the Hirshfeld Surface analysis. As for DHEA-GA, the higher proportion of H···O/O···H interactions (28.2%) indicates the strong H-bond interactions between DHEA-GA and aqueous solvent to some extent during the process of solvation, which promotes the improvement of solubility. With regard to DHEA-PHBA, the further precipitation after 60 min might also be another reason for its limited enhancement in solubility. When the coformer escapes from the lattice too quickly for its excellent solubility in the medium, the supersaturated DHEA will precipitate to give loosely aggregated clusters in some cases, along with the disappearance of superiority in solubility [38].

Besides, all PXRD results in Figure S6 indicate that the resulting powder samples of cocrystals are still in their cocrystal forms partly under these experiments except for the pH 6.8 condition. Under pH 6.8 buffer with 0.5% PVP K13-18, cocrystals were completely transformed into DHEA and coformers. It is speculated that weakly acidic coformer tends to be drawn out more quickly under neutral or basic environments than that under an acidic environment. In addition, DHEA transformed to its Form II (ZOYMOP01) under all these dissolution behavior experiments (Figure S7) [39].

### 3.8. Pharmacokinetic Analysis

**Figure 11:**
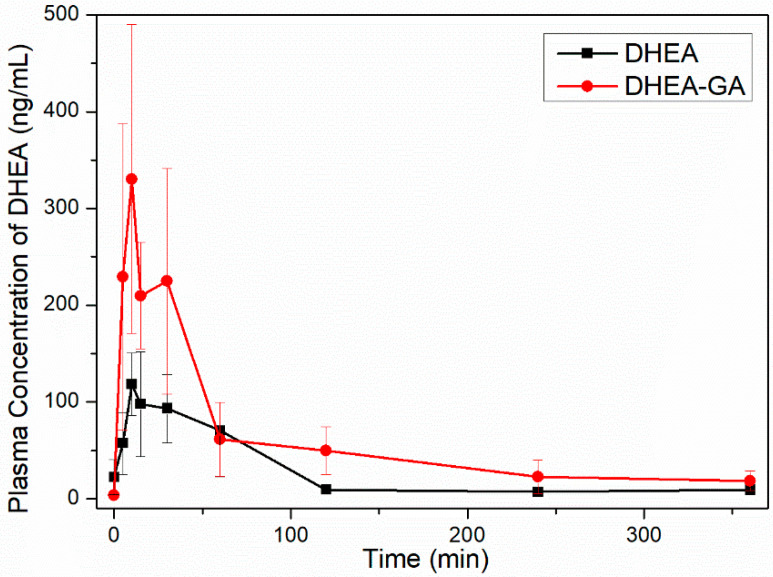
Plasma DHEA concentration versus time curves of DHEA and DHEA-GA in SD rats (male, *n* = 6).

Oral absorption of crystalline DHEA and its cocrystal was evaluated in male rats at 21 mg/kg. The profiles of plasma concentration versus time and correlative characteristic parameters (C_max_, AUC_0–t,_ and T_max_) are displayed in Figure 11 and Table 2. As observed in Figure 11, it is clear that DHEA-GA possesses a significant superiority over DHEA raw material with a higher maximum plasma concentration and enhanced bioavailability. The results show that the AUC_0–t_ of DHEA-GA is 2.2 times that of DHEA (*p* < 0.05), and the C_max_ of cocrystal is 2.8 times that of DHEA (*p* < 0.05). The enhancement of relative oral bioavailability may be attributed to enhanced solubility and adsorption of DHEA via creating the supersaturated state of DHEA molecules in the gastrointestinal tract. However, as the substrate of hepatic metabolic enzymes, DHEA is metabolized quickly and eliminated immediately with T_max_ at 10 min. The rapid adsorption process of DHEA-GA also results in the fading of the advantage in adsorption after 60 min.

## 4. Conclusions

In this study, eight cocrystals of DHEA were successfully prepared through multiple methods, and the single crystal structures were determined by SCXRD. To improve the solubility of DHEA, a functional food supplement used as an assisted reproductive sex hormone, eight new cocrystals of DHEA were designed and synthesized. These cocrystals share a common supramolecular motif C=O···H-O-Ar. Corresponding properties, including physicochemical properties, solubility, and dissolution performance in-vitro and in-vivo, were investigated in this paper. For these two edible cocrystals, DHEA-GA exhibits its superiority in solubility with approximately 2-fold enhancement in both pH 2.0 and pH 4.5 buffer solution. This superiority in in-vitro dissolution behavior is further reflected in its great enhancement in in-vivo bioavailability. The AUC of DHEA-GA was about 2.2 times that of pure DHEA. The advantages of DHEA-GA make it a promising alternative to pure DHEA to be developed in the future.

## Figures and Tables

**Table 1:** Calculated extreme MEPs value of DHEA and twelve coformers.

Compound	Maximum MEPs Value (kcal/mol)	Minimum MEPs Value (kcal/mol)
DHEA	38.74	**−39.19**
Gallic acid (GA)	**62.17**	−38.17
5-Hydroxyisophthalic acid (5HIPA)	**59.15**	−33.86
p-Hydroxybenzoic acid (PHBA)	**55.61**	−39.41
Pyrocatechol (CAT)	**54.80**	−28.36
Resorcinol (RES)	**50.09**	−27.41
Phloroglucinol (PG)	**49.96**	−28.06
1,5-Dihydroxy naphthalene (DHN)	**49.42**	−25.18
Hydroquinone (HQ)	**49.08**	−29.1
Anthranilic acid (AA)	**49.08**	−35.92
p-Aminophenol (PAP)	46.84	−31.13
p-Aminobenzoic acid (PABA)	43.59	−38.67
p-Phenylenediamine (PPDA)	31.43	−31.39

**Table 2:** Pharmacokinetic parameters of DHEA and DHEA-GA.

	DHEA	DHEA-GA
T_max_ (min)	10	10
C_max_ (ng·mL^−1^)	118.3 ± 32.4	330.4 ± 159.4 *
AUC_0–t_ (ng·min·mL^−1^)	9410.0 ± 1288.4	21,036.7 ± 4213.6 *

* *p*-value less than 0.05 represent a significant difference between the results of DHEA-GA and crystalline DHEA.

## Data Availability

The results obtained for all experiments performed are shown in the manuscript and Supplementary Materials, the raw data will be provided upon request.

## Supplementary Materials

The following supporting information can be downloaded at: https://www.mdpi.com/article/10.3390/pharmaceutics14112478/s1, Figure S1: Crystal packing mode of DHEA; Figure S2: Comparison between experimental and simulated PXRD patterns of DHEA cocrystals: (a) DHEA-CAT, (b) DHEA-HQ, (c) DHEA-RES, (d) DHEA-PG-DH, (e) DHEA-DHN, (f) DHEA-PHBA, (g) DHEA-GA, and (h) DHEA-5HIPA; Figure S3: DSC (blue line) and TGA (red line) curves of (a) DHEA-CAT, (b) DHEA-HQ, (c) DHEA-RES, (d) DHEA-PG-DH, (e) DHEA-DHN, (f) DHEA-PHBA, (g) DHEA-GA, and (h) DHEA-5HIPA; Figure S4: Comparison of DSC diagrams among DHEA, coformers, and corresponding cocrystals: (a) DHEA-CAT, (b) DHEA-HQ, (c) DHEA-RES, (d) DHEA-PG-DH, (e) DHEA-DHN, (f) DHEA-PHBA, (g) DHEA-GA, and (h) DHEA-5HIPA; Figure S5: FT-IR spectra of DHEA, coformers, and corresponding cocrystals: (a) DHEA-CAT, (b) DHEA-HQ, (c) DHEA-RES, (d) DHEA-PG-DH, (e) DHEA-DHN, (f) DHEA-PHBA, (g) DHEA-GA, and (h) DHEA-5HIPA; Figure S6: PXRD pattern for the resulting materials of (a) DHEA, (b) DHEA-GA, and (c) DHEA-PHBA after solubility and powder dissolution experiments, and (d) comparison of PXRD patterns among the resulting materials of DHEA, DHEA-GA and DHEA-PHBA after solubility test; Figure S7: (a) Comparison between experimental and simulated PXRD patterns of DHEA and DHEA Form I (ZOYMOP), and (b) comparison between simulated PXRD patterns of reported DHEA Form II (ZOYMOP01) and that of DHEA after different experiments; Figure S8: PXRD patterns of DHEA, anthranilic acid, and physical mixture after grinding; Table S1: Coformers selected in DHEA cocrystal screening experiment; Table S2: Crystallographic data and structure refinement parameters for new forms of DHEA; Table S3: List of H-bond lengths and angles for DHEA and DHEA cocrystals; Table S4: Specific melting points and fusion enthalpies of DHEA, coformers, and DHEA cocrystals; Table S5: Characteristic absorption band of C=O stretching vibration on DHEA; Table S6: Proportion of H-O/O-H interaction among multiple solid forms.
