# Supplementary material for: Dehydroepiandrosterone Cocrystals with Improved Solubility and Bioavailability
Source: Pharmaceutics. 2022 Nov 16;14(11):2478. doi: 10.3390/pharmaceutics14112478 (PMC9699216; doi:10.3390/pharmaceutics14112478)
Supplement: Supplementary file 1 [file pharmaceutics-14-02478-s001.zip › pharmaceutics-2011108-supplementary.pdf]

Dehydroepiandrosterone Cocrystals with Improved Solubility and Bioavailability

Yihua Jiang, [1,2] *Yinxiang Cheng* [1,2], *Mengyuan Xia* [1 2], *Bingrui Zhang* [1,2],

Qiaoce Ding [1], *Liye Lu,* [1] *Jian-Rong Wang* [1,*] *and Xuefeng Mei* [1,2,*]

[1]*Pharmaceutical Analytical & Solid-State Chemistry Research Center, Shanghai Institute of Materia Medica, Chinese Academy of Sciences, Shanghai 201203, China*

[2]*University of Chinese Academy of Sciences, No. 19A Yuquan Road, Beijing 100049, China*

Correspondence: jrwang@simm.ac.cn (J.-R.W.); xuefengmei@simm.ac.cn (X.M.)

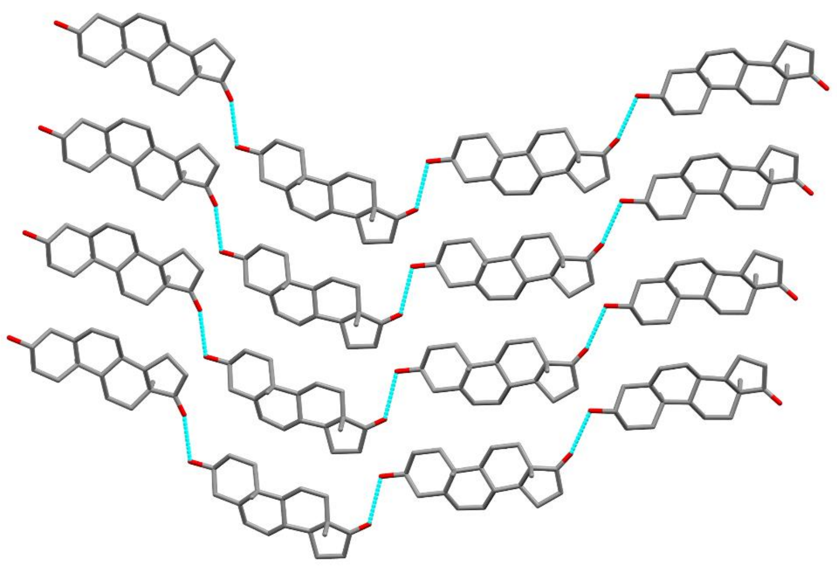

Figure S1. Crystal packing mode of DHEA.

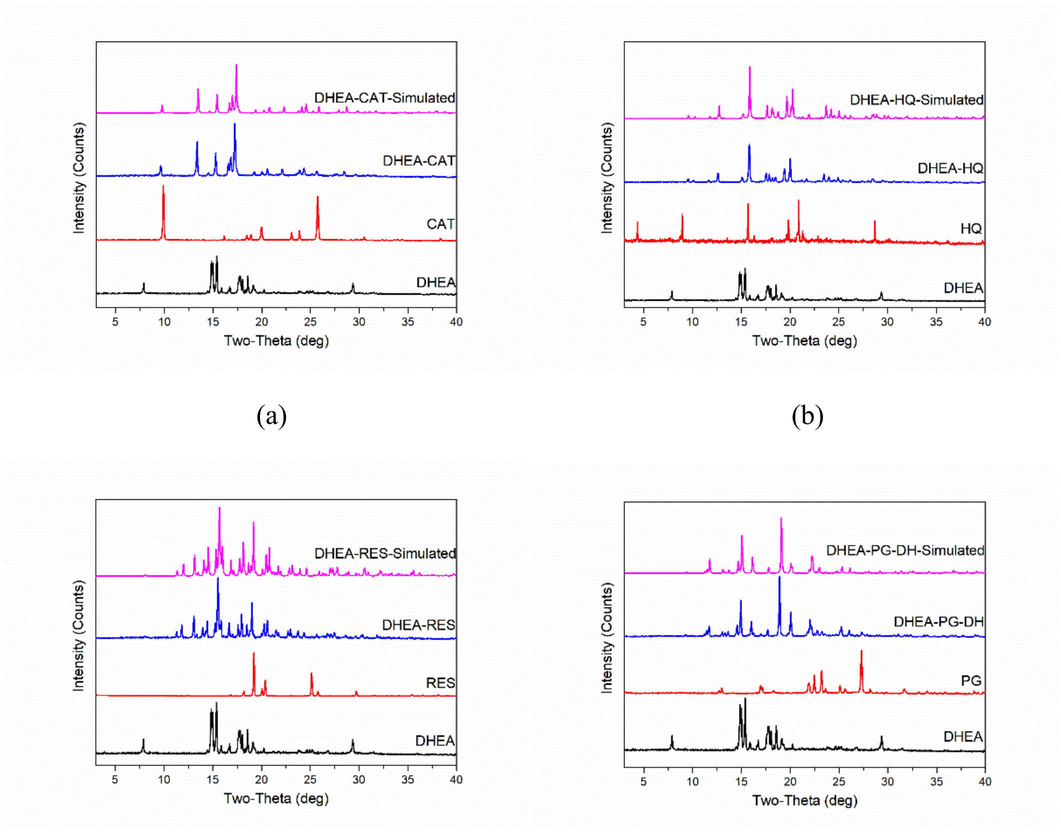

(a) (b)

(c) (d)

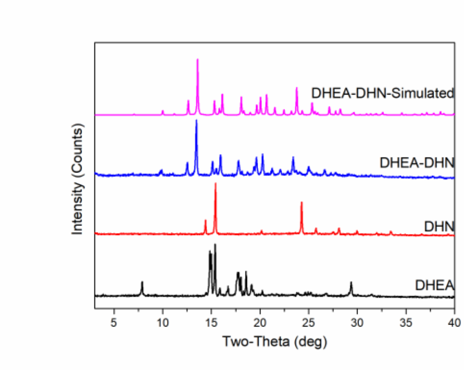

(e)

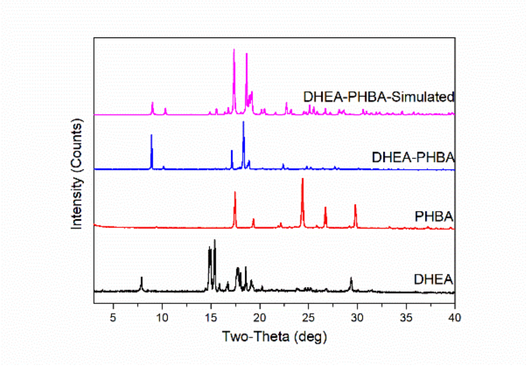

(f)

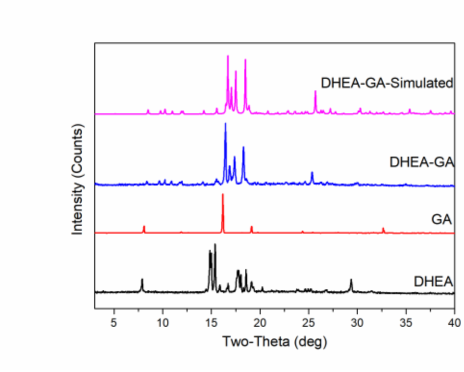

(g)

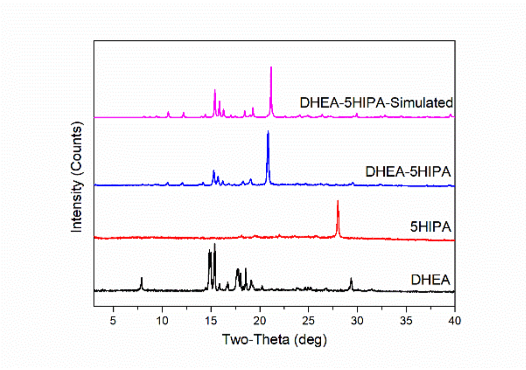

(h)

Figure S2. Comparison between experimental and simulated PXRD patterns of DHEA cocrystals: (a) DHEA-CAT, (b) DHEA-HQ, (c) DHEA-RES, (d) DHEA-PG-DH, (e) DHEA-DHN, (f) DHEA-PHBA, (g) DHEA-GA, and (h) DHEA-5HIPA.

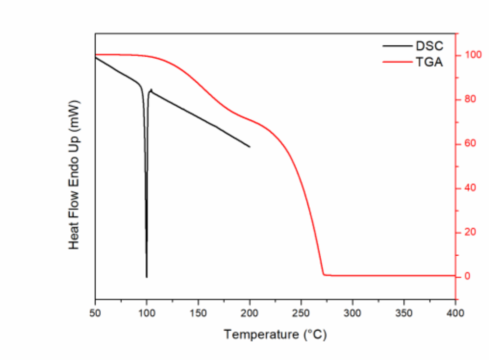

(a)

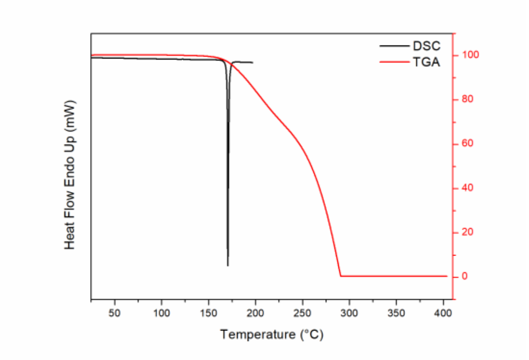

(b)

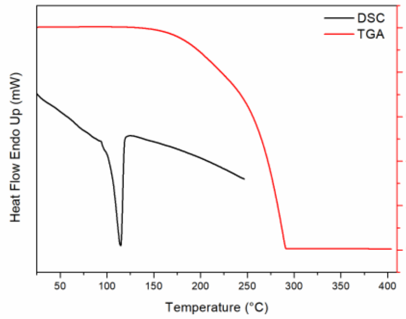

(c)

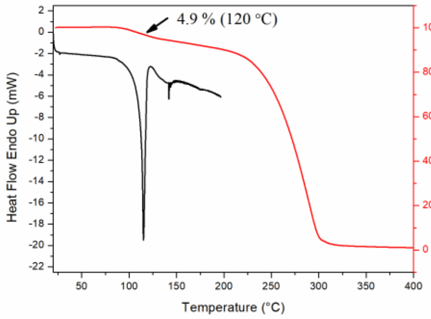

(d)

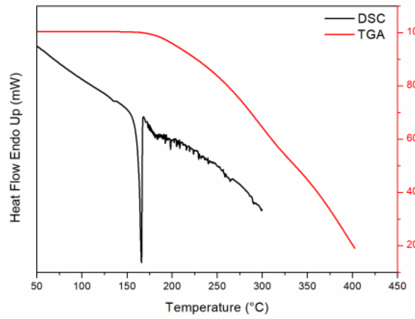

(e)

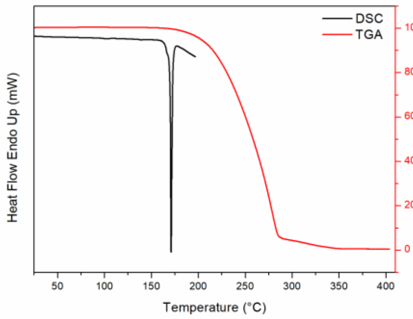

(f)

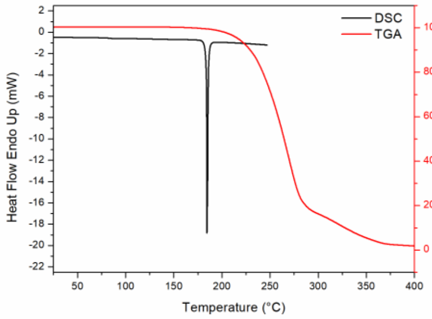

(g)

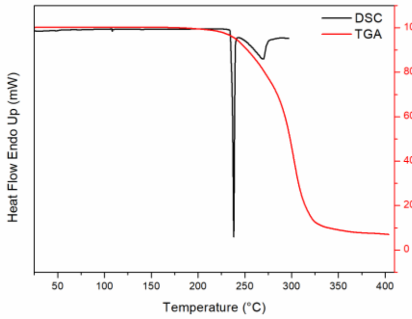

(h)

Figure S3. DSC (blue line) and TGA (red line) curves of (a) DHEA-CAT, (b) DHEA-HQ, (c) DHEA-RES, (d) DHEA-PG-DH, (e) DHEA-DHN, (f) DHEA-PHBA, (g) DHEA-GA, and (h) DHEA-5HIPA.

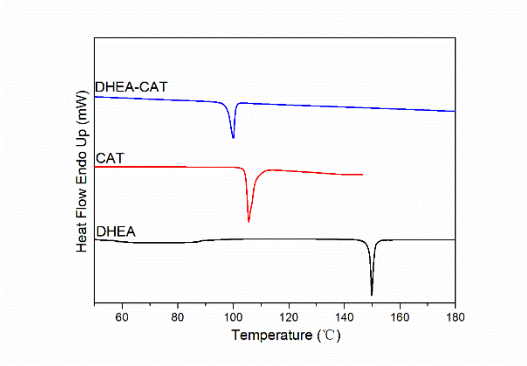
DHEA-CAT
CAT
DHEA
Heat Flow Endo Up (mW)
60 80 100 120 140 160 180
Temperature (℃)

(a)

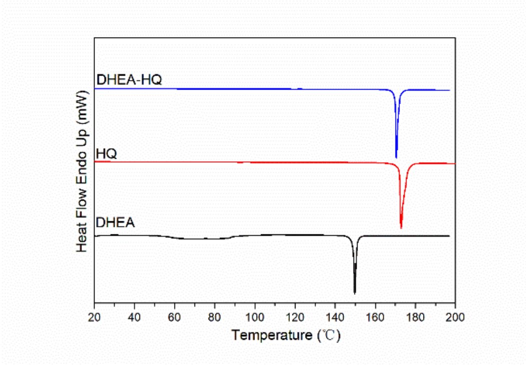
DHEA-HQ
HQ
DHEA
Heat Flow Endo Up (mW)
20 40 60 80 100 120 140 160 180 200
Temperature (℃)

(b)

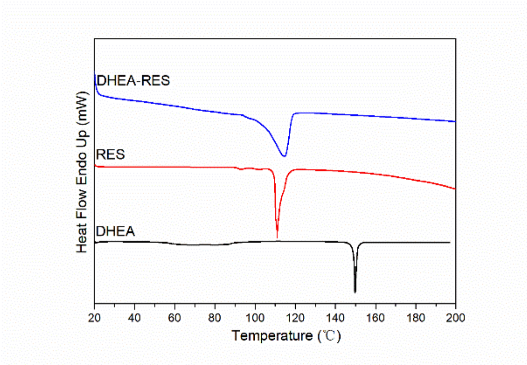
DHEA-RES
RES
DHEA
Heat Flow Endo Up (mW)
20 40 60 80 100 120 140 160 180 200
Temperature (℃)

(c)

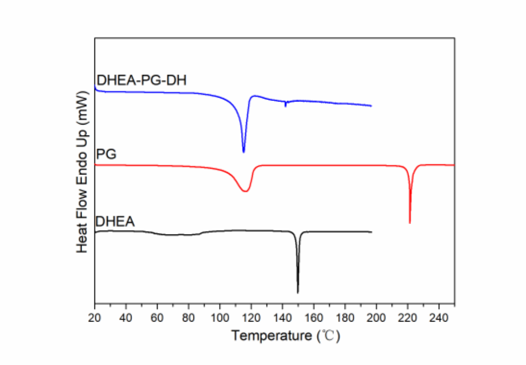
DHEA-PG-DH
PG
DHEA
Heat Flow Endo Up (mW)
20 40 60 80 100 120 140 160 180 200 220 240
Temperature (℃)

(d)

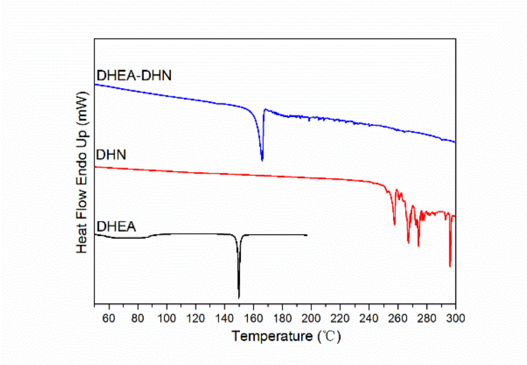
DHEA-DHN
DHN
DHEA
Heat Flow Endo Up (mW)
60 80 100 120 140 160 180 200 220 240 260 280 300
Temperature (℃)

(e)

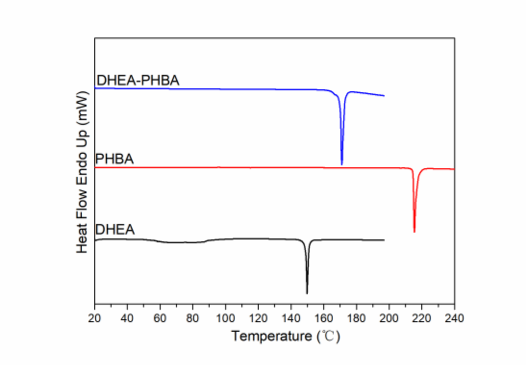
DHEA-PHBA
PHBA
DHEA
Heat Flow Endo Up (mW)
20 40 60 80 100 120 140 160 180 200 220 240
Temperature (℃)

(f)

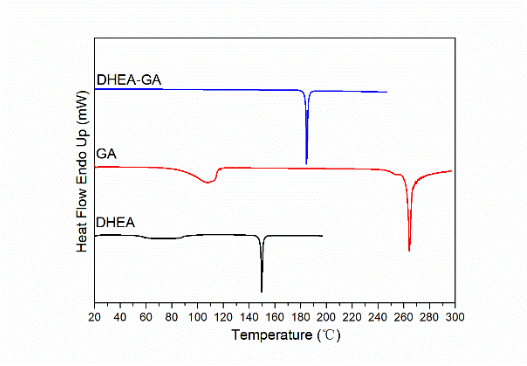
DHEA-GA
GA
DHEA
Heat Flow Endo Up (mW)
20 40 60 80 100 120 140 160 180 200 220 240 260 280 300
Temperature (℃)

(g)

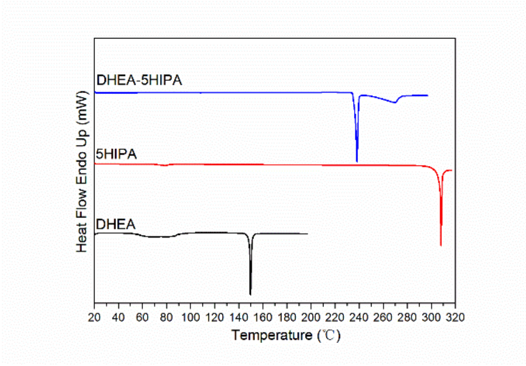
DHEA-5HIPA
5HIPA
DHEA
Heat Flow Endo Up (mW)
20 40 60 80 100 120 140 160 180 200 220 240 260 280 300 320
Temperature (℃)

(h)

Figure S4. Comparison of DSC diagrams among DHEA, coformers and corresponding cocrystals: (a) DHEA-CAT, (b) DHEA-HQ, (c) DHEA-RES, (d) DHEA-PG-DH, (e) DHEA-DHN, (f) DHEA-PHBA, (g) DHEA-GA, and (h) DHEA-5HIPA.

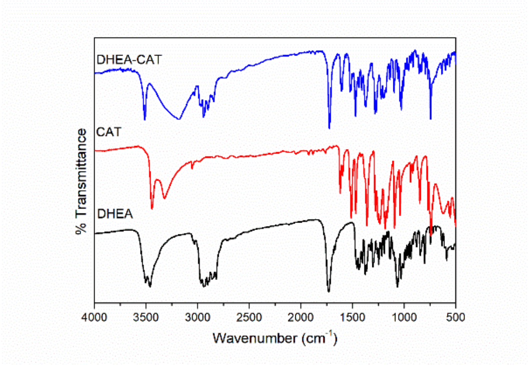

(a)

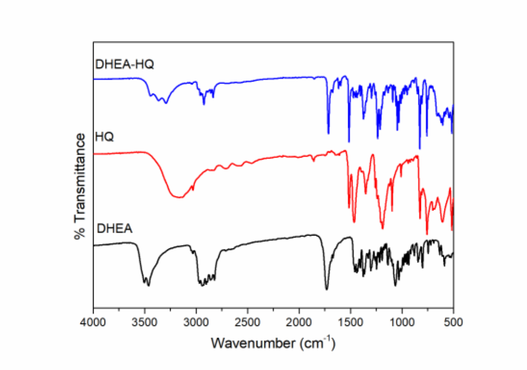

(b)

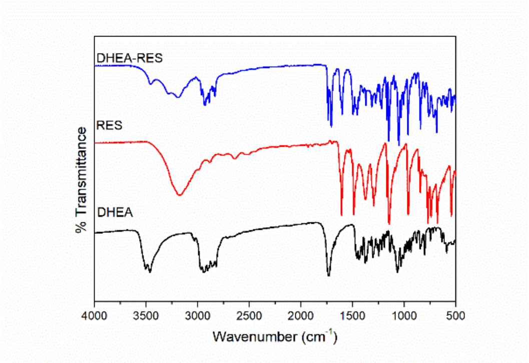

(c)

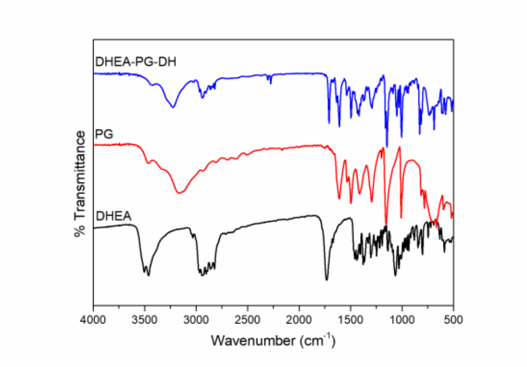

(d)

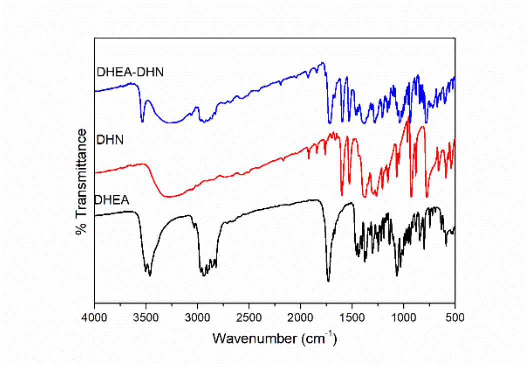

(e)

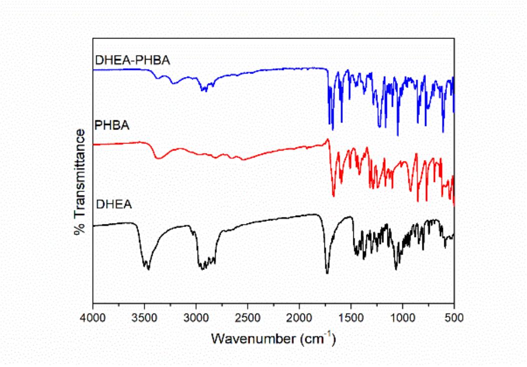

(f)

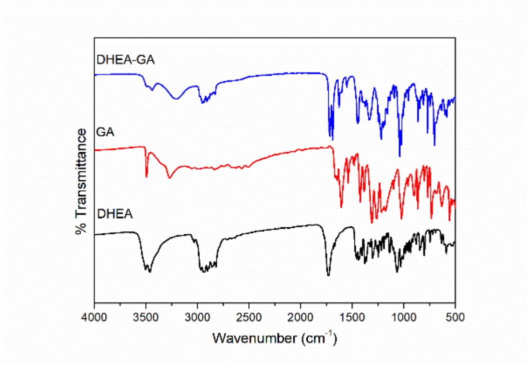

(g)

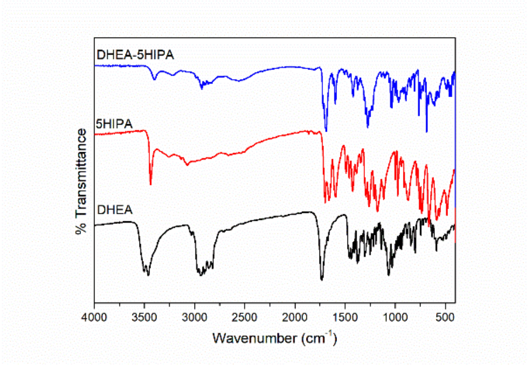

(h)

Figure S5. FT-IR spectra of DHEA, coformers and corresponding cocrystals: (a) DHEA-CAT, (b) DHEA-HQ, (c) DHEA-RES, (d) DHEA-PG-DH, (e) DHEA-DHN, (f) DHEA-PHBA, (g) DHEA-GA, and (h) DHEA-5HIPA.

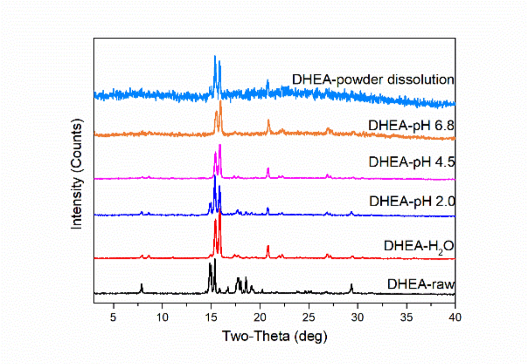

(a)

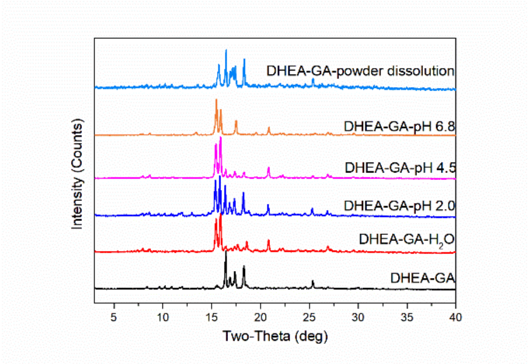

(b)

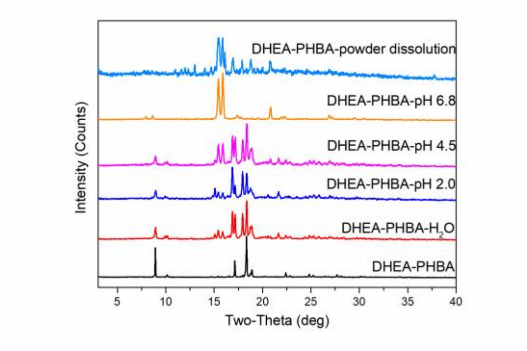

(c)

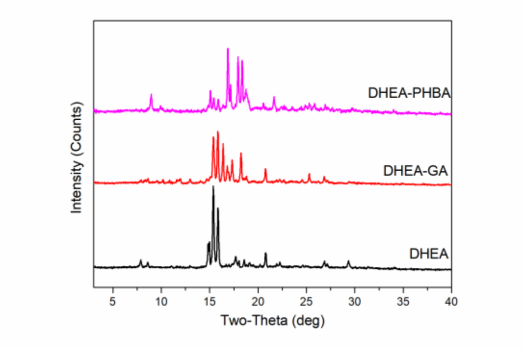

(d)

Figure S6. PXRD pattern for the resulting materials of (a) DHEA, (b) DHEA-GA, and (c) DHEA-PHBA after solubility and powder dissolution experiments, and (d): comparison of PXRD patterns among the resulting materials of DHEA, DHEA-GA and DHEA-PHBA after solubility test.

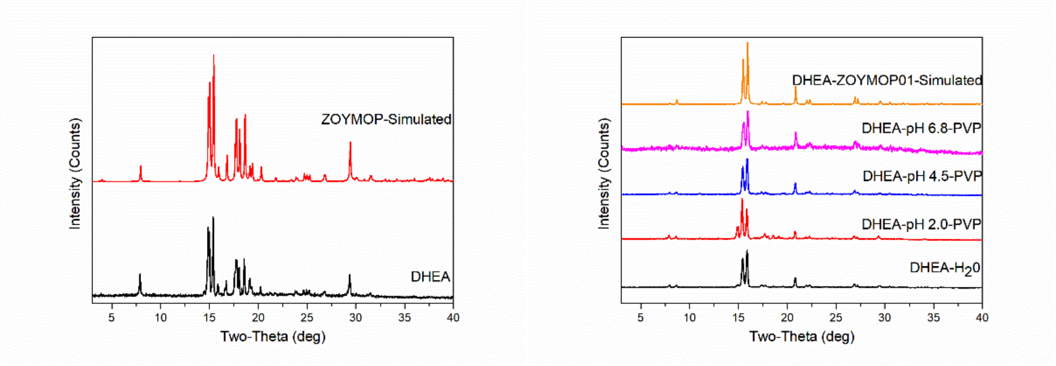

(a) (b)

Figure S7. (a) Comparison between experimental and simulated PXRD patterns of DHEA and DHEA Form I (ZOYMOP), and (b) comparison between simulated PXRD patterns of reported DHEA Form II (ZOYMOP01) and that of DHEA after different experiments.

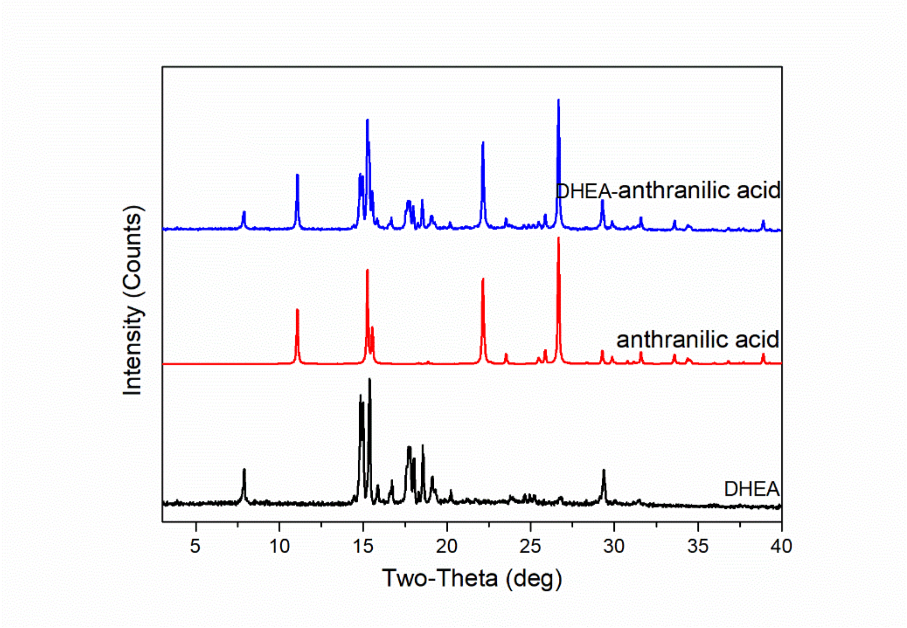

Figure S8. PXRD patterns of DHEA, anthranilic acid, and physical mixture after grinding.

Table S1. Coformers selected in DHEA cocrystal screening experiment.

NO.	Coformer	NO.	Coformer
1	**Gallic acid**	16	Methylparaben
2	**p-hydroxybenzoic acid**	17	3,5-dihydroxybenzoate
3	**Hydroquinone**	18	Trimesic acid
4	**Pyrocatechol**	19	2-Naphthol
5	**Resorcinol**	20	1-hydroxy-2-naphthonic acid
6	**1, 5-dihydroxynaphthalene**	21	5-Nitroisophthalic acid
7	**5-Hydroxyisophthalic acid**	22	4-Hydroxy-cinnamic acid
8	**Phloroglucinol**	23	3-hydroxy-benzoic acid

9	Anthranilic acid	24	Vanillic acid
10	p-Phenylenediamine	25	Phthalic acid
11	p-Aminobenzoic acid	26	Propyl gallate
12	p-Aminophenol	27	Terephthalic acid
13	o-Phenylenediamine	28	1-Naphthoic acid
14	5-Aminoisophthalic acid	29	Isophthalic acid
15	Methyl-3,5-dihydroxybenzoate	30	2-hydroxy-3-naphthonic acid

Table S2. Crystallographic data and structure refinement parameters for new forms of DHEA.

	**DHEA-CAT**	**DHEA-HQ**	**DHEA-RES**	**DHEA-PG-DH**
Formula	$C_{25}H_{34}O_4$	$C_{25}H_{34}O_4$	$C_{44}H_{62}O_6$	$C_{44}H_{66}O_9$
Crystal system	Orthorhombic	Orthorhombic	Orthorhombic	Monoclinic
Space group	$P2_12_12_1$	$P2_12_12_1$	$P2_12_12_1$	$P2_1$
Temperature(K)	170(2)	170(2)	170(2)	150(2)
a (Å)	6.3885(8)	9.7609(9)	6.5181(8)	7.8417(3)
b (Å)	18.080(2)	11.7034(11)	23.171(3)	26.5046(13)
c (Å)	19.087(3)	18.4429(19)	24.813(3)	9.4339(4)
α (deg)	90	90	90	90
β (deg)	90	90	90	90.150(6)
γ (deg)	90	90	90	90
V (Å^3)	2204.6(5)	2106.8(4)	3747.5(8)	1960.75(15)
D_{Cal} (g/cm^3)	1.201	1.256	1.218	1.252
Z	4	4	4	2
λ (Mo Kα)	0.7107	0.7107	0.7107	0.7107
Independent reflections	4402	4312	7653	7240
GOF	1.031	1.043	1.075	1.130

R_{int}	0.0637	0.0776	0.0832	0.0773
R_1	0.0630	0.0430	0.0552	0.0775
wR_2	0.1329	0.1009	0.1247	0.1921
	**DHEA-DHN**	**DHEA-PHBA**	**DHEA-GA**	**DHEA-5HIPA**
Formula	$C_{29}H_{36}O_4$	$C_{26}H_{34}O_5$	$C_{45}H_{62}O_9$	$C_{27}H_{34}O7$
Crystal system	Orthorhombic	Monoclinic	Monoclinic	Triclinic
Space group	$P2_12_12_1$	$P2_1$	$P2_1$	P_1
Temperature(K)	170(2)	170(2)	150(2)	170(2)
a (Å)	7.6269(5)	5.9728(4)	10.7510(4)	10.9892(15)
b (Å)	17.6512(15)	19.6177(13)	10.6906(4)	11.4290(14)
c (Å)	17.6980(13)	9.5547(6)	17.8607(6)	11.4576(15)
α (deg)	90	90	90	79.554(4)
β (deg)	90	95.737(2)	104.8110(10)	71.335(4)
γ (deg)	90	90	90	62.457(3)
V (Å^3)	2382.6(3)	1113.94(13)	1969.57(12)	1207.9(3)
D_{Cal} (g/cm^3)	1.251	1.272	1.259	1.294
Z	4	2	2	2
λ (Mo Kα)	0.7107	0.7107	0.7107	0.7107
Independent reflections	4834	4544	7973	8014
GOF	1.076	1.073	1.054	1.060
R_{int}	0.0534	0.0718	0.0496	0.0673
R_1	0.0457	0.0547	0.0441	0.0715

wR2	0.1002	0.1111	0.1038	0.1827

*the Z number represents the cocrystal formula in a unit cell

Table S3. List of H-bond lengths and angles for DHEA and DHEA cocrystals.

Form	interaction	D-H/Å	H…A/ Å	D…A/ Å	∠D-H…A/ Å	symmetry code
**DHEA-CAT**	O3-H3A…O2	0.85	1.93(6)	2.726(6)	155	-1/2+x, 1/2-y, -z
	O4-H4…O1	0.84	1.86(6)	2.692(6)	166	1/2-x, 1-y, -1/2+z
**DHEA-HQ**	O1-H1…O4	0.84(4)	2.05(4)	2.882(3)	175	1-x, 1/2+y, 3/2-z
	O4-H4…O2	0.84	2.00	2.833(3)	175	x, y, 1+z
**DHEA-RES**	O1-H1…O3	0.92(3)	1.95(3)	2.859(4)	171	-x, 1/2+y, 3/2-z
	O3-H3A…O5	0.94(4)	1.94(4)	2.858(4)	167	1-x, -1/2+y, 3/2-z
**DHEA-PG**	O1-H1…O2	0.84	2.49	3.001(7)	120	1-x, 1/2+y, 2-z
	O6-H6A…O3	0.84	1.96	2.755(7)	159	1+x, y, z
	O6-H6A…O4	0.84	2.56	2.943(7)	109	1-x, -1/2+y, 1-z
	O8-H8A…O3	0.87	2.12	2.905(7)	150	1+x, y, z
	O8-H8B…O2	0.87	2.13	2.950(7)	157	1-x, 1/2+y, 2-z
	O9-H9A…O8	0.87	1.95	2.781(9)	160	-1+x, y, -1+z
	O9-H9B…O6	0.87	1.99	2.850(7)	167	-1+x, y, z
**DHEA-DHN**	O4-H4…O1	0.90(4)	1.85(4)	2.739(3)	169	1+x, -1+y, z
**DHEA-PHBA**	O1-H1…O3	0.92(7)	2.02(7)	2.893(5)	157	-1+x, y, z
	O5-H5…O2	0.84	1.91	2.731(4)	164	x, 1+y, z
**DHEA-GA**	O1-H1…O5	0.84	1.97	2.764(3)	158	x, y, -1+z
	O3-H3…O8	0.81(5)	2.28(5)	2.984(3)	147	-1+x, y, z
	O7-H7…O4	0.84	1.92	2.721(3)	159	1+x, y, 1+z
	O8-H8A…O1	0.84	1.99	2.726(3)	146	1+x, y, 1+z
**DHEA-5HIPA**	O1-H1…O11	0.84	1.90	2.731(4)	170	x, -1+y, z
	O3-H3A…O7	0.85	1.86	2.701(4)	167	1+x, y, z
	O6-H6A…O12	0.91	1.71	2.602(4)	168	-1+x, y, z
	O8-H8A…O1	0.86	1.74	2.597(5)	170	x, y, 1+z
	O9-H9A…O4	0.84	1.93	2.766(5)	171	-1+x, 1+y, 1+z
	O10-H10…O3	0.85	1.71	2.548(4)	165	-1+x, 1+y, -1+z
	O13-H13…O5	0.88	1.71	2.587(4)	179	1+x, y, z
	O14-H14A…O2	0.84	1.92	2.753(5)	171	x, y, -1+z

Table S4. Specific melting points and fusion enthalpies of DHEA, coformers and DHEA cocrystals.

Compound	Melting point (°C)	Fusion enthalpy (J/g)	Compound	Melting point (°C)	Fusion enthalpy (J/g)
DHEA	149.34	84.68±10.00	DHEA-CAT	101.90	78.86±10.00
CAT	172.60	174.90±20.00	DHEA-HQ	170.10	128.2±15.00
HQ	172.24	254.10±35.00	DHEA-RES	118.48	52.61±10.00
RES	109.86	401.77±60.00	DHEA-PG-DH	112.51	125.8±20.00

PG	104.44	554.30±60.00	DHEA-DHN	168.25	93.88±10.00
DHN	260.77	89.57±15.00	DHEA-PHBA	170.2	112.0±15.00
PHBA	214.67	231.0±20.00	DHEA-GA	184.22	110.22±15.00
GA	263.62	403.1±60.00	DHEA-5HIPA	236.59	90.63±10.00
5HIPA	307.74	240.4±30.00			

Table S5. Characteristic absorption band of C=O stretching vibration on DHEA

Compound	Wavenumbers (cm^{-1})
DHEA	1731.00
DHEA-CAT	1720.00
DHEA-HQ	1713.28
DHEA-RES	1704.73
DHEA-PG-DH	1707.65
DHEA-DHN	1716.09
DHEA-PHBA	1709.18
DHEA-GA	1716.82
DHEA-5HIPA	1715.56

Table S6. Proportion of H-O/O-H interaction among multiple solid forms.

Compound	Proportion of H-O/O-H interactions
DHEA	16.4 %
DHEA-CAT	16.4 %
DHEA-HQ	18.9 %
DHEA-RES	19.1 %
DHEA-PG-DH	15.3 %
DHEA-DHN	15.0 %
DHEA-PHBA	23.5 %
DHEA-GA	28.2 %
DHEA-5HIPA	1715.56
